# Brain Kynurenine Pathway Metabolite Levels May Reflect Extent of Neuroinflammation in ALS, FTD and Early Onset AD

**DOI:** 10.3390/ph16040615

**Published:** 2023-04-19

**Authors:** Annelies Heylen, Yannick Vermeiren, Ido P. Kema, Martijn van Faassen, Claude van der Ley, Debby Van Dam, Peter P. De Deyn

**Affiliations:** 1 Laboratory of Neurochemistry and Behavior, Experimental Neurobiology Unit, University of Antwerp, 2610 Antwerp, Belgium; 2 Division of Human Nutrition and Health, Chair Group of Nutritional Biology, Wageningen University and Research, 6708 Wageningen, The Netherlands; 3 Faculty of Medicine & Health Sciences, Translational Neurosciences, University of Antwerp, 2000 Antwerp, Belgium; 4 Department of Laboratory Medicine, University of Groningen, University Medical Center Groningen, 9713 Groningen, The Netherlands; 5 Department of Neurology and Alzheimer Center Groningen, University of Groningen, University Medical Center Groningen, 9713 Groningen, The Netherlands

**Keywords:** amyotrophic lateral sclerosis, frontotemporal dementia, early onset Alzheimer’s disease, anthranilic acid, kynurenic acid, quinolinic acid, biomarker, mass spectrometry, LC-MS/MS

## Abstract

Objectives: Despite distinct clinical profiles, amyotrophic lateral sclerosis (ALS) and frontotemporal dementia (FTD) patients share a remarkable portion of pathological features, with a substantial percentage of patients displaying a mixed disease phenotype. Kynurenine metabolism seems to play a role in dementia-associated neuroinflammation and has been linked to both diseases. We aimed to explore dissimilarities in kynurenine pathway metabolites in these early onset neurodegenerative disorders in a brain-region-specific manner. Methods: Using liquid chromatography mass spectrometry (LC-MS/MS), kynurenine metabolite levels were determined in the brain samples of 98 healthy control subjects (n = 20) and patients with early onset Alzheimer’s disease (EOAD) (n = 23), ALS (n = 20), FTD (n = 24) or a mixed FTD–ALS (n = 11) disease profile. Results: Overall, the kynurenine pathway metabolite levels were significantly lower in patients with ALS compared to FTD, EOAD and control subjects in the frontal cortex, substantia nigra, hippocampus and neostriatum. Anthranilic acid levels and kynurenine-to-tryptophan ratios were consistently lower in all investigated brain regions in ALS compared to the other diagnostic groups. Conclusions: These results suggest that the contribution of kynurenine metabolism in neuroinflammation is lower in ALS than in FTD or EOAD and may also be traced back to differences in the age of onset between these disorders. Further research is necessary to confirm the potential of the kynurenine system as a therapeutic target in these early onset neurodegenerative disorders.

## 1. Introduction

Frontotemporal dementia (FTD) and amyotrophic lateral (ALS) are both predominantly early onset and invariably fatal neurodegenerative disorders. FTD is considered one of the major causes of early onset dementia apart from early onset Alzheimer’s disease (EOAD) [1]. As the name indicates, the main structures affected in FTD are the frontal and temporal lobes, often to a varying extent. This results in a wide range of behavioral and cognitive disturbances, such as inappropriate and disinhibited behaviors and difficulties with executive functioning and affective syndromes, as well as language dysfunction.

Behavioral changes are usually the first symptoms to appear, whereas memory and perceptual spatial skills are initially unaffected. In later stages, patients may also start to develop motor deficits [2]. FTD is part of a broader class of frontotemporal lobar degeneration or FTLD. In general, FTLD is divided into three types, depending on the predominant symptoms: behavioral variant frontotemporal dementia, semantic variant primary progressive aphasia and non-fluent variant primary progressive aphasia [3,4]. The first type makes up nearly 50% of all FTD cases and the term “FTD” often refers to this form [4]. Thus far, there are no definitive fluid FTD biomarkers, and diagnostic criteria rely solely on the presence of behavioral and cognitive symptoms in combination with neuroimaging. Possible behavioral variant FTD requires the presence of at least three out of six behavioral and cognitive features, namely (1) apathy or inertia (lack of emotion or concern), (2) behavioral disinhibition, (3) loss of empathy (lack of sympathizing with emotions of others), (4) perseverative/compulsive behaviors, (5) hyperorality and (6) a dysexecutive neuropsychological profile with relative sparing of memory and visuospatial skills [3,5,6]. A probable FTD diagnosis requires a typical clinical FTD profile with superimposed functional decline and imaging changes in frontotemporal structures, e.g., alterations in brain metabolism (as measured through PET or SPECT) or frontal lobe atrophy (as measured by CT or MRI scans). A definite diagnosis is only possible upon postmortem histopathological examination or in the case of confirmed genetic mutations when an FTD diagnosis is already suspected [5,6].

ALS is a progressive motor neuron disease, leading to upper motor neuron signs (spasticity and hyperreflexia), lower motor neuron signs (muscle weakness and atrophy) and weight loss [7,8]. Based on the El Escorial criteria, an ALS diagnosis requires evidence of progressive upper and lower motor neuron deficits in a minimum of one limb or body region or lower motor neuron deficits confirmed by clinical examination (one region) and/or by an electromyogram (EMG) in two body regions (defined as bulbar, cervical, thoracic and lumbosacral) [8].

Although both disorders seem to have distinct clinical profiles, mounting evidence suggests they share multiple clinical, genetic and pathological features. FTD has been linked to ALS in approximately 10–15% of patients [3,4,9]. Clinical ALS signs such as spasticity, hyperreflexia, muscle weakness and atrophy or Parkinsonism have been seen in half of FTD patients, whereas frontotemporal-related cognitive and behavioral disturbances have been observed in up to 40–50% of ALS patients [3,9]. A vast number of genetic studies seem to support this link. Aggregation of transactive response DNA binding protein with a molecular weight of 43 kDa (*TDP-43*) has been observed in the majority of both FTD and ALS cases [10]. The common hexanucleotide GGGGCC repeat expansion mutation in the chromosome 9 open reading frame 72 (*C9orf72*) gene and the loss-of-function mutation in TANK-binding kinase (*TBK1*) have been recently identified as major actors in both FTD and ALS pathology [11,12]. Fused in sarcoma (*FUS*)-based inclusions account for a smaller percentage of FTD cases, but have also been linked to ALS [3,13]. Some research suggests that microtubule-associated protein tau (*MAPT*) or shortly Tau mutations, which are known to cause a specific FTD phenotype called FTD-Tau, may be responsible for part of the clinical profile of some ALS cases as well [14,15].

To date, there are no American Food and Drug Administration (FDA)- or European Medicines Agency (EMA)-approved treatments for FTD. In addition, currently prescribed drugs are mostly psychotropic, based on monoaminergic system manipulations, and solely rely on symptomatic relief [16]. These drugs have been developed for the treatment of various neurological and psychiatric disorders and may have moderate success; however, none of these are disease specific and tailored to the neurochemical profile of FTD. Up until now, there are two FDA-approved treatment options for ALS. Riluzole is a glutamate receptor antagonist; however, it provides only modest improvements in survival rates (with an estimated two to four months) [17]. Edavarone is a free radical scavenger, for which several studies report a delay in motor decline based on the revised ALS functional rating scale (ALSFRS-R) [18,19]. On the other hand, both drugs have been associated with a wide range of side effects, whereas they have not been proven to modify the disease course of ALS [20,21]. There are several ongoing clinical trials with promising drug candidates for ALS. Although not disease modifying, Nuedexta, an FDA-approved kinidine/dextromethorphan combination drug, seems to have a positive effect on speech, swallowing and salivation in bulbar ALS [22]. MN-166 (Ibudilast) is reported to improve ALSFRS-R and ALS assessment questionnaire-5 items (ALSAQ-5) scores, as well as the average muscle strength in ALS patients [23], whereas treatment with masitinib was associated with a survival benefit of up to 25 months in patients with early diagnosed ALS [24]. Significant improvements in ALSFRS-R scores and survival has been seen in ALS patients treated with AMX0035 (phenylbutyrate/tauroursodeoxycholic acid combination drug). Its use for the treatment of ALS has been conditionally approved in Canada as of June 2022, with approval in the United States and Europe also pending [25,26,27]. However, at this moment, effective and approved disease-modifying treatments are still lacking.

**Figure 1:**
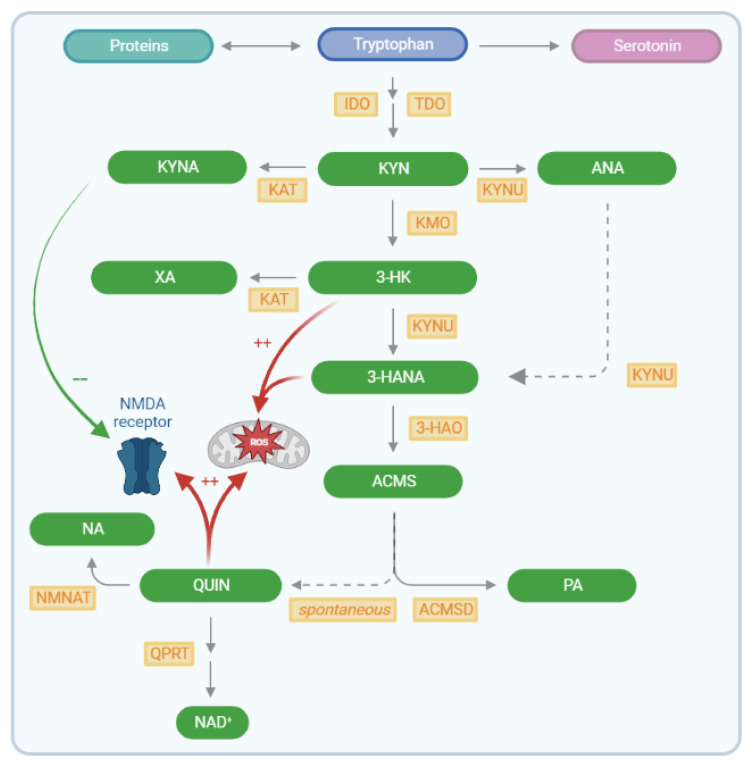
Schematic overview of the kynurenine metabolic pathway. The kynurenine metabolic pathway starts with the ingestion of TRP through diet. Via the uptake by large neutral amino acid transporters, TRP crosses the blood–brain barrier [47]. From here on, TRP is either used for protein synthesis, in the minor serotonergic metabolic pathway or for the formation of kynurenine and its metabolites. KYN can either be catalyzed to ANA, KYNA or 3-HK. In turn, 3-HK is then either catalyzed into XA or to 3-hydroxyanthranilic acid (3-HANA) and ACMS, an intermediate product which then results in spontaneous conversion to QUIN or metabolization to PA. Eventually, QUIN is metabolized to NA or NAD^+^. The kynurenine pathway leads to the formation of three neuroactive compounds: the neuroprotective KYNA (NMDA receptor antagonist) in astrocytes and the formation of neurotoxic compounds 3-HK, 3-HANA (both generate ROS) and QUIN (NMDA receptor agonist and generates ROS) in microglia [32,33]. Abbreviations: TRP = tryptophan, KYN = kynurenine, ANA = anthranilic acid, KYNA = kynurenic acid, 3-HK = 3-hydrokynurenine, XA = xanthurenic acid, 3-HANA = 3-hydroxyanthranilic acid, ACMS = 2-amino-3-carboxymuconate 6-semialdehyde, QUIN = quinolinic acid, PA = picolinic acid, NA = nicotinic acid, NAD^+^ = nicotinamide-adenine-dinucleotide, IDO = indoleamine 2,3-dioxygenase, TDO = tryptophan 2,3-dioxygenase, KYNU = kynureninase, KAT = kynurenine aminotransferase, KMO = kynurenine 3-monooxygenase, 3-HAO = 3-hydroxyanthranillic acid dioxygenase, ACMSD = 2-amino-3-carboxymuconate 6-semialdehyde decarboxylase, QPRT = quinolinate phosporibosyltransferase, NMNAT = nicotinamide mononucleotide adenylyl transferase, NMDA = N-methyl-D-aspartate, ROS = reactive oxygen species. Image created with BioRender and adapted from Schwieler et al. [48].

The inefficacy of current treatments stimulates research in other directions. For example, the etiology of FTD–ALS may also be driven by the process of neuroinflammation. Neuroinflammation is considered one of the major hallmarks of multiple neurodegenerative diseases, as it may exacerbate the pathological formation and accumulation of toxic proteins [28]. One route via which neuroinflammation could aggravate neurodegeneration involves the (over)activation of the kynurenine pathway (KP), the major route of tryptophan (TRP) catabolism (see Figure 1) [29,30,31]. This leads to the formation of four neuroactive compounds. In astrocytes, the KP is favored towards the formation of the neuroprotective kynurenic acid (KYNA), an N-methyl-D-aspartate (NMDA) receptor antagonist. In microglia, the formation of neurotoxic compounds 3-hydroxykynurenine (3-HK), 3-hydroxyanthranilic acid (3-HANA) and quinolinic acid (QUIN) is favored. All three compounds promote the formation of reactive oxygen species, while QUIN acts as a potent NMDA receptor agonist as well [32,33]. KP alterations have already been established in Alzheimer’s disease (AD), but have been less extensively studied in FTD and ALS. Interestingly, we recently observed a significant decrease in the serum 3-HK/xanthurenic acid (XA) ratio in ALS compared to FTD patients, although no other KP compounds were significantly changed in cerebrospinal fluid (CSF) or serum [34]. Several other studies found significant differences in KYNA levels between AD and Parkinson’s disease versus controls [35], and between ALS and controls for KYNA [36], L-kynurenine (KYN), TRP and the indoleamine 2,3-dioxygenase (IDO) index [33]. There is one study that suggests a generally increased brain kynurenine metabolism in AD patients compared to controls, with pronounced KYNA elevations in the putamen and caudate nucleus [37]. In the past few years, inhibitors of IDO and tryptophan 2,3-dioxygenase (TDO) have been put forward as potential therapeutic targets. For example, TDO inhibition was found to be neuroprotective and to improve cognitive function in mouse, fruit fly and worm models of AD and Parkinson’s disease, presumably through activation reduction of the KP [38,39,40,41]. In addition, inhibiting the kynurenine metabolic pathway might also favor the minor serotonergic metabolic pathway and increase serotonin turnover. This could positively affect patients since multiple neurodegenerative disorders are associated with decreased serotonergic activity [42,43,44].

It is important to keep in mind that KP compounds diffuse over the blood–brain barrier and blood–CSF barrier to varying degrees, and that diffusion can be influenced by the integrity of these barriers. For instance, TRP, KYN, 3-HK and anthranilic acid (ANA) seem to be freely transported, while QUIN, 3-HANA and KYNA diffuse rather poorly [45,46]. This might explain why some kynurenine metabolites are seemingly unaltered in CSF or blood. Hence, we cannot exclude the possibility of central and/or peripheral KP alterations in AD, FTD or ALS. To date, there are no studies examining KP compounds in EOAD or in different brain regions of ALS or FTD. Therefore, this route requires more thorough investigation. 

In recent decades, there has been a growing awareness that FTD and ALS are no distinct disease entities, but rather intertwine in some sort of spectrum. On one end, patients can present with pure frontotemporal degeneration with no motor deficits, on the other end, there are patients who merely experience motor neuron symptoms, with a wide continuum of patients in between that experience both FTD- and ALS-associated symptoms to varying degrees. There has been increasing evidence for a role of the kynurenine metabolic pathway in the progress of ALS and FTD [29,30,31,34,35,36,37]. Furthermore, both disease-modifying and effective symptomatic treatments for the two diseases are still lacking and there is a high need for novel drug targets. The purpose of our study was to further clarify (dis)similarities in kynurenine neurotransmission between FTD and ALS patients, whilst also including EOAD patients and healthy control individuals as a positive control for early onset disease and negative reference, respectively. A subgroup of mixed FTD–ALS patients was included as well. Overall, the characterization of disease-specific kynurenine profiles for FTD, ALS or FTD–ALS could not only increase our knowledge of the FTD–ALS spectrum, but also contribute to the identification of novel immunological targets of interest, as well as predictive or diagnostic biomarkers. To this end, we have analyzed KP compounds in brain samples of patients with ALS, FTD, mixed FTD–ALS and EOAD and of control individuals.

## 2. Results

### 2.1. Demographic Information

A summary of demographic information and medication use can be found in Table 1. In total, we included 98 subjects with FTD (n = 24), ALS (n = 20) and FTD–ALS (n = 11) phenotypes, in addition to EOAD patients (n = 23) and control individuals (n = 20), as a positive and negative control group, respectively. The age at death of subjects varied from 38 to 83 years old with a mean age of 66 years (66.6 ± 8.6 years). The male to female ratio was 52 to 46. Subjects were sex-matched for each group (X²(4, N = 98) = 5.488, *p* > 0.05), but the median age at death significantly differed between all diagnostic categories. Pairwise group comparisons with a Fisher’s Exact Test showed that age at death was significantly lower for ALS patients than for FTD, FTD–ALS patients and control individuals; however, only the latter comparison remained significant (two-tailed *p* = 0.048) after adjusting significance values for multiple comparisons. Medication records were collected for most of the patients. The classes of drugs relevant for this study are listed in Table 1. This includes psychotropic medication, hormones and other CNS-acting drugs, since they are intimately linked with the kynurenine system. Overall, the number of subjects taking versus not taking psychotropic medication significantly differed (two-tailed *p* = 0.004). We found similar differences in the use of antidepressants (two-tailed *p* = 0.003), antipsychotics (two-tailed *p* = 0.012), CNS-acting medication (two-tailed *p* < 0.001), antidementia drugs (two-tailed *p* = 0.023) and adrenergic medication (two-tailed *p* = 0.029). Only comparisons remaining significant following Bonferroni correction (*p* ≤ 0.005 for ten pairwise comparisons) are described here. Antidepressant and antipsychotic use were significantly higher in EOAD patients compared to CONTR subjects (two-tailed *p* = 0.005 and *p* = 0.005, respectively). The use of CNS-acting drugs was significantly higher in ALS compared to FTD, EOAD and CONTR subjects (two-tailed *p* < 0.001, *p* < 0.001 and *p* < 0.001, respectively). Lastly, no between-group differences were found for adrenergic medication.

### 2.2. Kynurenine Pathway Findings 

**Figure 2:**
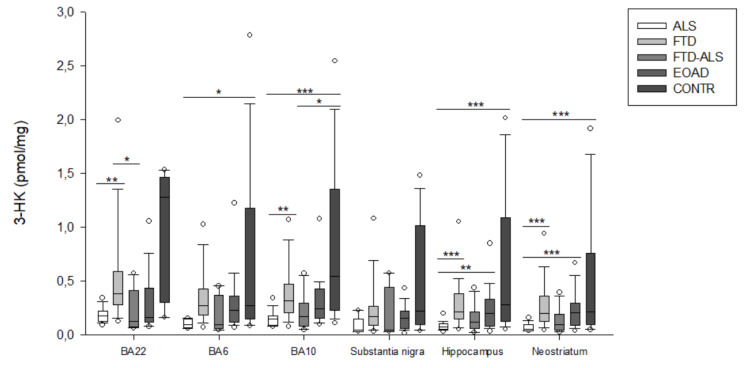
3-HK levels of ALS, FTD, FTD–ALS, EOAD and CONTR participants. 3-HK levels are visualized by boxplots clustered according to brain region. White dots represent the minimal and maximal outliers. Post hoc Bonferroni-adjusted significance values are classified as *p* ≤ 0.05, *p* ≤ 0.01 and *p* ≤ 0.001 and are indicated by one, two or three asterisks, respectively. Abbreviations: BA22 = Brodmann area 22, ALS = amyotrophic lateral sclerosis, FTD = frontotemporal dementia, EOAD = early onset Alzheimer’s disease, CONTR = control individuals, 3-HK = 3-hydroxykynurenine.

**Figure 3:**
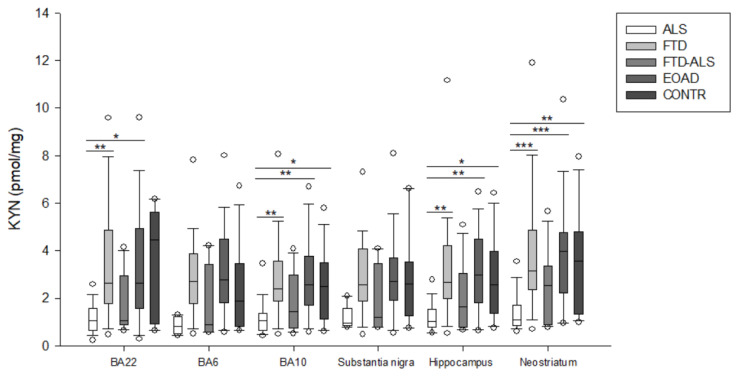
KYN levels of ALS, FTD, FTD–ALS, EOAD and CONTR participants. KYN levels are visualized by boxplots clustered according to brain region. White dots represent the minimal and maximal outliers. Post hoc Bonferroni-adjusted significance values are classified as *p* ≤ 0.05, *p* ≤ 0.01 and *p* ≤ 0.001 and are indicated by one, two or three asterisks, respectively. Abbreviations: BA22 = Brodmann area 22, ALS = amyotrophic lateral sclerosis, FTD = frontotemporal dementia, EOAD = early onset Alzheimer’s disease, CONTR = control individuals, KYN = kynurenine.

**Figure 4:**
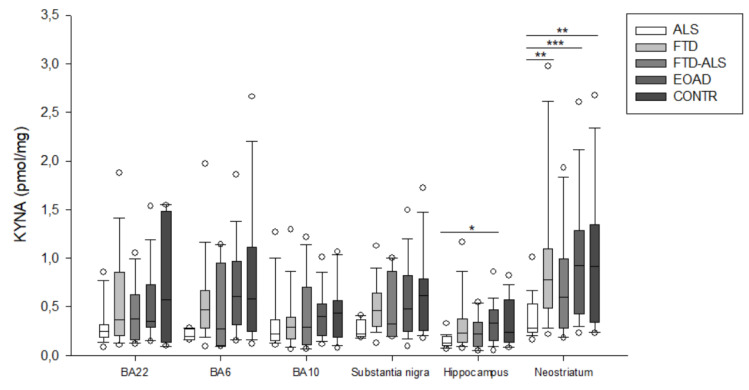
KYNA levels of ALS, FTD, FTD–ALS, EOAD and CONTR participants. KYNA levels are visualized by boxplots clustered according to brain region. White dots represent the minimal and maximal outliers. Post hoc Bonferroni-adjusted significance values are classified as *p* ≤ 0.05, *p* ≤ 0.01 and *p* ≤ 0.001 and are indicated by one, two or three asterisks, respectively. Abbreviations: BA22 = Brodmann area 22, ALS = amyotrophic lateral sclerosis, FTD = frontotemporal dementia, EOAD = early onset Alzheimer’s disease, CONTR = control individuals, KYNA = kynurenic acid.

**Figure 5:**
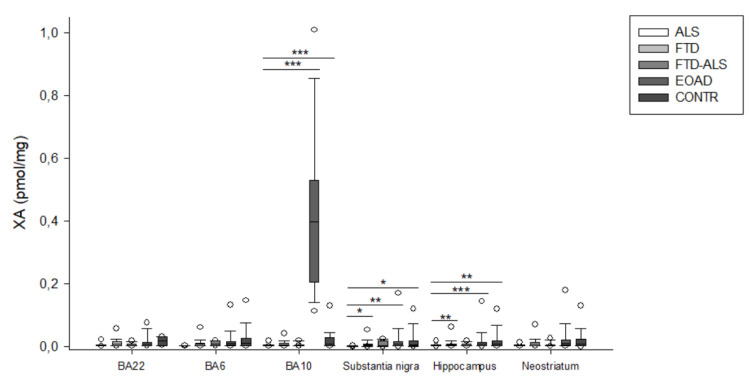
XA levels of ALS, FTD, FTD–ALS, EOAD and CONTR participants. XA levels are visualized by boxplots clustered according to brain region. White dots represent the minimal and maximal outliers. Post hoc Bonferroni-adjusted significance values are classified as *p* ≤ 0.05, *p* ≤ 0.01 and *p* ≤ 0.001 and are indicated by one, two or three asterisks, respectively. Abbreviations: BA22 = Brodmann area 22, ALS = amyotrophic lateral sclerosis, FTD = frontotemporal dementia, EOAD = early onset Alzheimer’s disease, CONTR = control individuals, XA = xanthurenic acid.

**Figure 6:**
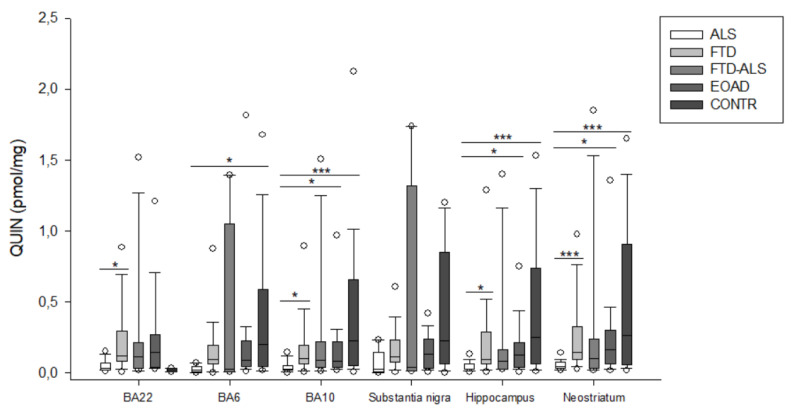
QUIN levels of ALS, FTD, FTD–ALS, EOAD and CONTR participants. QUIN levels are visualized by boxplots clustered according to brain region. White dots represent the minimal and maximal outliers. Post hoc Bonferroni-adjusted significance values are classified as *p* ≤ 0.05, and *p* ≤ 0.001 and are indicated by one or three asterisks, respectively. Abbreviations: BA22 = Brodmann area 22, ALS = amyotrophic lateral sclerosis, FTD = frontotemporal dementia, EOAD = early onset Alzheimer’s disease, CONTR = control individuals, QUIN = quinolinic acid.

**Figure 7:**
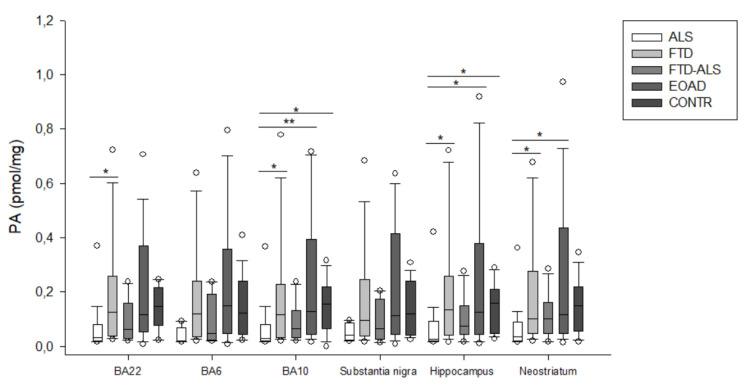
PA levels of ALS, FTD, FTD–ALS, EOAD and CONTR participants. PA levels are visualized by boxplots clustered according to brain region. White dots represent the minimal and maximal outliers. Post hoc Bonferroni-adjusted significance values are classified as *p* ≤ 0.05 and *p* ≤ 0.01 and are indicated by one or two asterisks, respectively. Abbreviations: BA22 = Brodmann area 22, ALS = amyotrophic lateral sclerosis, FTD = frontotemporal dementia, EOAD = early onset Alzheimer’s disease, CONTR = control individuals, PA = picolinic acid.

**Figure 8:**
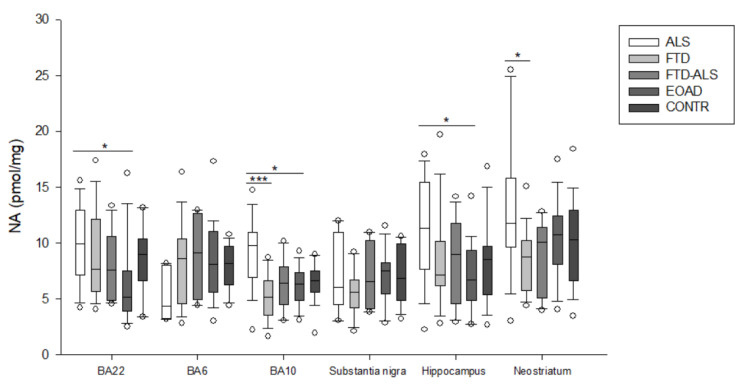
NA levels of ALS, FTD, FTD–ALS, EOAD and CONTR participants. NA levels are visualized by boxplots clustered according to brain region. White dots represent the minimal and maximal outliers. Post hoc Bonferroni-adjusted significance values are classified as *p* ≤ 0.05 and *p* ≤ 0.001 and are indicated by one or three asterisks, respectively. Abbreviations: BA22 = Brodmann area 22, ALS = amyotrophic lateral sclerosis, FTD = frontotemporal dementia, EOAD = early onset Alzheimer’s disease, CONTR = control individuals, NA = nicotinic acid.

**Figure 9:**
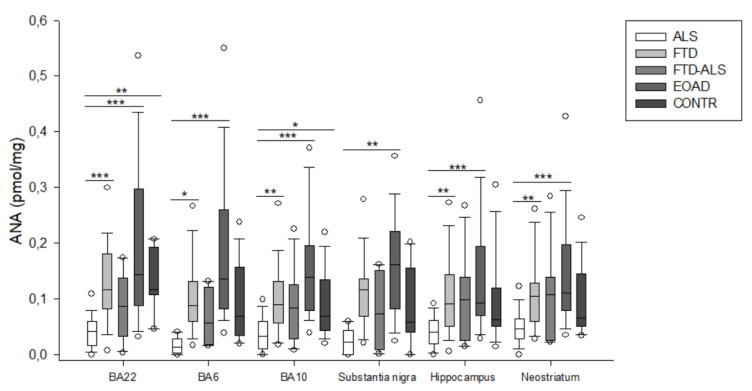
ANA levels of ALS, FTD, FTD–ALS, EOAD and CONTR participants. ANA levels are visualized by boxplots clustered according to brain region. White dots represent the minimal and maximal outliers. Post hoc Bonferroni-adjusted significance values are classified as *p* ≤ 0.05, *p* ≤ 0.01 and *p* ≤ 0.001 and are indicated by one, two or three asterisks, respectively. Abbreviations: BA22 = Brodmann area 22, ALS = amyotrophic lateral sclerosis, FTD = frontotemporal dementia, EOAD = early onset Alzheimer’s disease, CONTR = control individuals, ANA = anthranilic acid.

**Figure 10:**
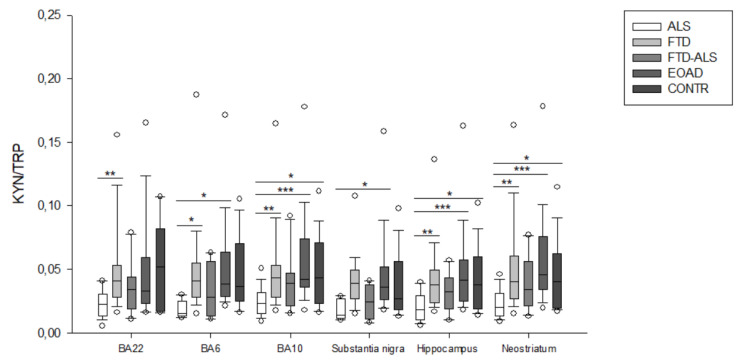
KYN/TRP ratios of ALS, FTD, FTD–ALS, EOAD and CONTR participants. KYN/TRP ratios are visualized by boxplots clustered according to brain region. White dots represent the minimal and maximal outliers. Post hoc Bonferroni-adjusted significance values are classified as *p* ≤ 0.05, *p* ≤ 0.01 and *p* ≤ 0.001 and are indicated by one, two or three asterisks, respectively. Abbreviations: BA22 = Brodmann area 22, ALS = amyotrophic lateral sclerosis, FTD = frontotemporal dementia, EOAD = early onset Alzheimer’s disease, CONTR = control individuals, KYN = kynurenine, TRP = tryptophan.

Brain KP metabolite levels for all groups are listed in Table 2 and Appendix A, Table A1, Table A2, Table A3, Table A4 and Table A5. Due to the high number of significant comparisons, *p*-values for pairwise comparisons are not mentioned in the text, but indicated in Appendix A, Table A1, Table A2, Table A3, Table A4 and Table A5, by means of superscript letters, and in Figure 2, Figure 3, Figure 4, Figure 5, Figure 6, Figure 7, Figure 8, Figure 9 and Figure 10 by asterisks.

Firstly, in BA22, we found significant differences between the five diagnostic categories for 3-HK (*p* < 0.001) (Figure 2), KYN (*p* = 0.001) (Figure 3), XA (*p* = 0.001) (Figure 5), QUIN (*p* < 0.001) (Figure 6), PA (*p* = 0.005) (Figure 7), NA (*p* = 0.030) (Figure 8), ANA levels (*p* < 0.001) (Figure 9) and the KYN/TRP ratio (*p* = 0.005) (Figure 10). A post hoc analysis with Bonferroni correction revealed significantly decreased levels of 3-HK, KYN, QUIN, PA, ANA levels and the KYN/TRP ratio in ALS compared to FTD patients. We detected similar differences between ALS and EOAD patients for KYN and ANA levels, and between ALS patients and CONTR individuals for ANA levels. Initial differences in XA, QUIN levels and the KYN/TRP ratio between ALS versus EOAD or CONTR subjects were no longer significant after applying Bonferroni correction. Ultimately, we also found that BA22 3-HK levels were significantly higher in FTD patients than in FTD–ALS patients. This was the only significant difference between these two patient groups in our entire analysis. Although not all remained significant upon multiple comparison correction, we identified similar decreases in KP levels between ALS compared to FTD, EOAD and CONTR subjects in all other brain regions. 

For example, in BA6, a KW analysis revealed significant differences for 3-HK (*p* = 0.017) (Figure 2), KYN (*p* = 0.042) (Figure 3), QUIN (*p* = 0.028) (Figure 6), ANA levels (*p* < 0.001) (Figure 9) and the KYN/TRP ratio (*p* = 0.027) (Figure 10). Pairwise comparisons revealed that ANA levels and the KYN/TRP ratio were significantly lower in ALS than in FTD and EOAD patients, as were 3-HK and QUIN levels compared to control individuals. 

In BA10, a KW analysis identified significant differences in 3-HK (*p* < 0.001) (Figure 2), KYN (*p* < 0.001) (Figure 3), XA (*p* < 0.001) (Figure 3), QUIN (*p* < 0.001) (Figure 6), PA (*p* = 0.003) (Figure 7), NA (*p* < 0.001) (Figure 8), ANA levels (*p* < 0.001) (Figure 9) and the KYN/TRP ratio (*p* < 0.001) (Figure 10). KYN, 3-HK, XA, QUIN, PA, ANA levels and the KYN/TRP ratio were all systematically and significantly lower in ALS than in FTD, EOAD or CONTR subjects, although differences in 3-HK in ALS versus EOAD and XA levels in ALS versus FTD were discarded following Bonferroni correction. BA10 3-HK levels were also significantly lower in FTD–ALS patients compared to control individuals.

In the substantia nigra, following overall differences in ANA levels (*p* = 0.003) (Figure 9) and the KYN/TRP ratio (*p* = 0.001) (Figure 10), we found that these levels were significantly lower in ALS compared to EOAD and FTD patients, although differences between ALS and FTD patients were no longer significant upon Bonferroni correction. 

KW analysis also revealed significant differences in hippocampal KP levels, including 3-HK (*p* < 0.001) (Figure 2), KYN (*p* < 0.001) (Figure 3), KYNA (*p* = 0.012) (Figure 4), XA (*p* = 0.002) (Figure 5), QUIN (*p* < 0.001) (Figure 6), PA (*p* = 0.006) (Figure 7), NA (*p* = 0.036) (Figure 8), ANA levels (*p* < 0.001) (Figure 9) and the KYN/TRP ratio (*p* < 0.001) (Figure 10). Upon pairwise analysis, we noted that 3-HK, KYN, XA, QUIN, PA levels and the KYN/TRP ratio were significantly lower in ALS than in FTD, EOAD or control subjects, as were KYNA levels in ALS versus EOAD patients and ANA levels in ALS compared to both FTD and EOAD patients.

Lastly, the outcome was very similar in the neostriatum, with significant differences for 3-HK (*p* < 0.001) (Figure 2), KYN (*p* < 0.001) (Figure 3), KYNA (*p* < 0.001) (Figure 4), QUIN (*p* < 0.001) (Figure 6), PA (*p* = 0.010) (Figure 7), NA (*p* = 0.030) (Figure 8), ANA levels (*p* < 0.001) (Figure 9) and the KYN/TRP ratio (*p* < 0.001) (Figure 10). When comparing KP levels group-to-group, we observed significantly lower neostriatal 3-HK, KYN, KYNA, XA, QUIN, PA, ANA levels and KYN/TRP ratios in ALS compared to FTD, EOAD and control subjects, although differences in PA and ANA levels did not remain significant following multiple comparison correction between ALS and control subjects. In contrast to all other kynurenine components, NA levels were generally higher in ALS than in FTD or EOAD subjects. NA levels were significantly higher in ALS compared to FTD or EOAD patients in BA10 and the neostriatum, whereas we also detected significant increases in ALS compared to EOAD patients in BA22, BA10 and the hippocampus. Interestingly, in BA6, NA levels were also lower in ALS patients compared to other groups, although this was not significant. We did not find significant differences in TRP levels or the 3-HK/XA ratio between the five groups in any of the brain regions.

The patients in our analyses were stratified by clinical and neuropathological diagnoses. Since there are differences within the scoring methods (A/B/C for Thal phase, Braak stage and CERAD score, respectively; Brettschneider; TDP-43), we also investigated KP levels across neuropathological stages within diagnostic groups. Here, we found that KYN/TRP ratios in BA10 and the substantia nigra (*p* = 0.036 and *p* = 0.018, respectively) and 3-HK/XA ratios in BA10 and the neostriatum (*p* = 0.014 for both) were significantly higher in FTD patients with Braak stage 1 or 2 versus those with Braak stage 3 or 4 pathology. However, these changes in the KYN/TRP ratio were no longer significant after applying Bonferroni correction. BA22 ANA levels were significantly lower in ALS patients having Thal phase 0 versus those having Thal phase 1 or 2 pathology (*p* = 0.016), which did not remain significant following Bonferroni correction.

### 2.3. Correlations to Psychotropic Medication and Demographic Factors

To investigate the potential interaction effect with psychotropic medication, we repeated our analysis including only subjects free of psychotropic medication. Firstly, in the overall study population, we found that there were no significant kynurenine differences between patients taking or not taking psychotropic medication in any of the brain regions, with the exception of BA6 QUIN levels, which were significantly higher in patients taking psychotropic medication (*p* = 0.043). After repeating the pairwise analyses while only including patients not taking psychotropic medication, we did not detect any differences either. Hippocampal ANA levels were significantly lower in EOAD compared to FTD/ALS patients; however, they surpassed the significance level upon post hoc correction. Following the significant differences in CNS-acting medication intake between diagnostic groups, we also performed a regression analysis to examine a possible role of CNS-acting drugs on KP metabolite levels. This revealed that taking CNS-acting medication negatively affected ANA levels in all brain regions [BA22 (*R*² = 0.145, *F*(1, 43) = 7.265, *p* = 0.010), BA6 (*R*² = 0.093, *F*(1, 57) = 5.818, *p* = 0.019), BA10 (*R*² = 0.131, *F*(1, 60) = 9.081, *p* = 0.004), substantia nigra (*R*² = 0.094, *F*(1, 58) = 6.011, *p* = 0.017), hippocampus (*R*² = 0.081, *F*(1, 57) = 5.015, *p* = 0.029), neostriatum (*R*² = 0.087, *F*(1, 61) = 5.839, *p* = 0.019)], as well as the hippocampal 3-HK/XA ratio (*R*² = 0.077, *F*(1, 57) = 4.774, *p* = 0.033). However, when only including ALS and FTD/ALS patients—the only groups taking CNS-acting medication—no such effects were observed.

**Figure 11:**
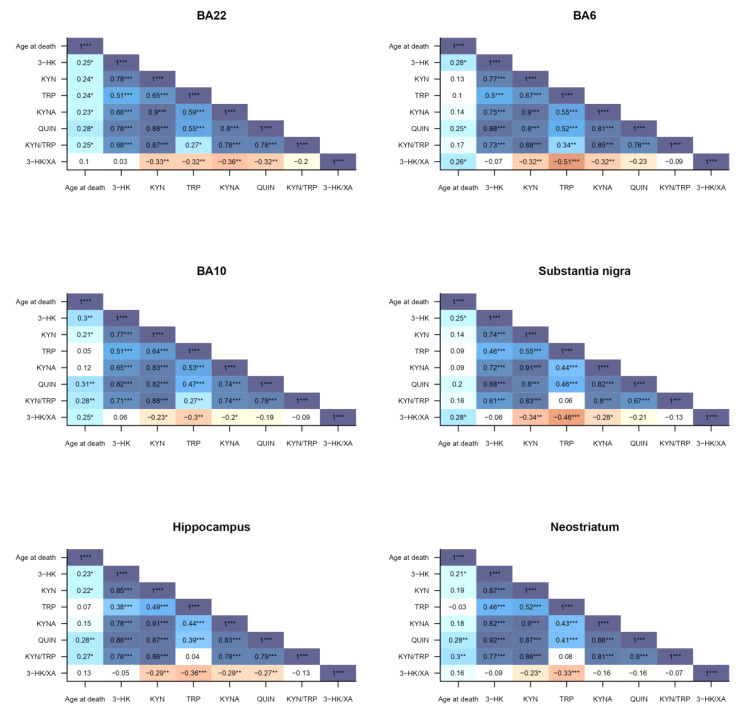
Correlations between age at death and KP levels of ALS, FTD, FTD–ALS, EOAD and CONTR participants. The correlation strength is visualized by color shade and correlation coefficients. Significance values are classified as *p* ≤ 0.05, *p* ≤ 0.01 and *p* ≤ 0.001 and are indicated by one, two or three asterisks, respectively. Abbreviations: KP = kynurenine pathway, BA22 = Brodmann area 22, BA6 = Brodmann area 6, BA10 = Brodmann area 10, ALS = amyotrophic lateral sclerosis, FTD = frontotemporal dementia, EOAD = early onset Alzheimer’s disease, CONTR = control individuals, 3-HK = 3-hydroxykynurenine, KYN = kynurenine, TRP = tryptophan, KYNA = kynurenic acid, QUIN = quinolinic acid, XA = xanthurenic acid.

There were significantly positive correlations between age at death and BA22 3-HK (*r_s_*(76) = 0.251, *p* = 0.029), BA22 KYN (*r_s_*(76) = 0.238, *p* = 0.038), BA22 TRP (*r_s_*(76) = 0.237, *p* = 0.039), BA22 KYNA (*r_s_*(76) = 0.230, *p* = 0.045), BA22 QUIN (*r_s_*(76) = 0.282, *p* = 0.013), BA22 KYN/TRP (*r_s_*(76) = 0.248, *p* = 0.031), BA6 3-HK (*r_s_*(68) = 0.279, *p* = 0.021), BA6 QUIN (*r_s_*(68) = 0.248, *p* = 0.042), BA6 3-HK/XA (*r_s_*(68) = 0.264, *p* = 0.029), BA10 3-HK (*r_s_*(96) = 0.298, *p* = 0.003), BA10 KYN (*r_s_*(96) = 0.214, *p* = 0.036), BA10 QUIN (*r_s_*(96) = 0.310, *p* = 0.002), BA10 KYN/TRP (*r_s_*(96) = 0.283, *p* = 0.005), BA10 3-HK/XA (*r_s_*(96) = 0.246, *p* = 0.016), substantia nigra 3-HK/XA (*r_s_*(67) = 0.275, *p* = 0.026), hippocampal 3-HK (*r_s_*(89) = 0.225, *p* = 0.034), hippocampal KYN (*r_s_*(89) = 0.218, *p* = 0.010), hippocampal QUIN (*r_s_*(89) = 0.280, *p* = 0.008), hippocampal KYN/TRP (*r_s_*(89) = 0.271, *p* = 0.010), neostriatal 3-HK (*r_s_*(97) = 0.213, *p* = 0.036), neostriatal QUIN (*r_s_*(97) = 0.28065, *p* = 0.005) and neostriatal KYN/TRP (*r_s_*(97) = 0.301, *p* = 0.003) levels (Figure 11). We additionally performed a linear regression analysis to investigate the effect of age at death on KP metabolite levels. This unraveled the significant effects of QUIN, NA and the 3-HK/XA ratio in multiple brain regions. Increased age was significantly associated with increases in QUIN levels in BA22 (*R*² = 0.095, *F*(1,74) = 7.740, *p* = 0.007), BA10 (*R*² = 0.057, *F*(1, 94) = 5.645, *p* = 0.020), the hippocampus (*R*² = 0.065, *F*(1, 87) = 6.012, *p* = 0.016) and neostriatum (*R*² = 0.070, *F*(1, 95) = 7.120, *p* = 0.009). We observed similar increases with age for the 3-HK/XA ratio in BA6 (*R*² = 0.078, *F*(1, 66) = 5.549, *p* = 0.021) and BA10 (*R*² = 0.068, *F*(1, 94) = 6.829, *p* = 0.010). In contrast, age had a significant inverse relation effect on NA levels, with NA levels decreasing with age (*R*² = 0.047, *F*(1, 94) = 4.648, *p* = 0.034). We also investigated the effect of sex on kynurenine differences. This revealed that there were some significant differences between KP levels of male and female study subjects. The KYN/TRP ratio was significantly higher in the BA22, BA10 and hippocampus of male subjects compared to female subjects (*p* = 0.020, *p* = 0.031 and *p* = 0.049, respectively). Hippocampal QUIN levels were also significantly higher in male than in female individuals (*p* = 0.038).

## 3. Discussion

Kynurenine-related neuroinflammation was initially studied in the context of AD, but to a lesser extent in ALS and FTD or EOAD. Most kynurenine research in FTD or ALS has been conducted on CSF or blood samples, whereas there are no studies reporting brain KP alterations in FTD or ALS. We measured the levels of KP compounds in frontal (BA10), temporal (BA22 and hippocampus) and motor-associated areas (BA6, substantia nigra and neostriatum)—all regions relevant to the pathology of the FTD–ALS spectrum—in order to unravel possible (dis)similarities between FTD and ALS patients. With the exception of NA levels, which were generally higher in the ALS group in all brain regions but BA6, we found that the levels of kynurenine compounds were consistently lower in ALS patients compared FTD, EOAD or control subjects in all examined brain regions.

In general, these findings suggest that kynurenine-associated neuroinflammation might not play a central role in ALS compared to other neurodegenerative disorders that are generally considered early onset, such as FTD and EOAD. However, when comparing KP levels between FTD or EOAD and control subjects, we did not detect any differences. This may suggest that kynurenine-related neuroinflammation is not necessarily specific for early onset neurodegenerative disorders. Moreover, the underlying cause of this striking difference between ALS and FTD, EOAD or control subjects might be found somewhere else. Although the average age in the ALS group was not substantially different from that in the other groups (ALS: 63.5, FTD: 67.6, FTD–ALS: 71.0, EOAD: 67.0, CONTR: 69.0), included ALS patients were significantly younger compared to their FTD, EOAD or control counterparts. This could explain why KP levels and in extension kynurenine-related neuroinflammation might be lower in our ALS patients, since aging is a primary risk factor for neuroinflammation, as well as neurodegenerative diseases in general [49]. The age at death was also significantly lower in ALS compared to FTD–ALS patients. However, we did not see any significant difference in KP levels between these two groups. This contradicts the idea that aging is unequivocally followed by an increase in KP levels. To further investigate this, we correlated age at death with brain KP levels. Although we found that levels of some kynurenine compounds were positively correlated with age, many others were negatively correlated and there was no consistency between age and specific kynurenine compounds or brain regions. On the other hand, a linear regression analysis to investigate the effect of age at death on KP metabolite levels revealed that increased age positively affected QUIN levels in BA22, BA10, the hippocampus and neostriatum, as well as the neurotoxic 3-HK/XA ratio in BA6 and BA10, which would be consistent with a neurotoxicity hypothesis of 3-HK and QUIN [32,33]. There was a significant increase in hippocampal QUIN levels and the KYN/TRP ratio in BA22, BA10 and hippocampus of male compared to female subjects, consistent with the earlier literature describing reduced KYN/TRP ratios and overall decreased KP activity in women compared to men [50,51,52,53]. This might be a consequence of interplay between the KP and hormonal regulation [51]. For example, several studies report interactions between the female hormones estradiol and estrogen with KP enzymes such as tryptophan oxygenase and vitamin B6-dependent enzymes such as kynureninase and quinolinic acid decarboxylase [54,55,56]. Nevertheless, we only noted sparse differences in KP compounds between men and women, which were not consistent throughout the entire KP nor between different regions.

Except for 3-HK levels in BA22, which were significantly higher in FTD in comparison to FTD–ALS patients, we could not detect any other differences between FTD or ALS patients with FTD–ALS patients. This suggests that there is little difference in KP activity, which could be a consequence of the overlapping pathology between FTD–ALS and the pure forms on both ends of the spectrum. However, in that case, we would not expect KP alterations between FTD and ALS patients. It raises the interesting question of whether these shared pathological features might differ between FTD and FTD–ALS and between ALS and FTD–ALS patients. ALS is a motor neuron disease in which the pathology initiates in the periphery and eventually spreads to (sub)cortical areas [57,58]. This might lead to higher neuroinflammation in the spinal cord compared to the brain, which could explain why KP levels were lower in brain regions of ALS patients. Moreover, Chen et al. found that IDO and QUIN presence was significantly higher in the spinal cord of ALS patients compared to control individuals [30]. Our results also raise another point, namely that all kynurenine compound levels are lower in ALS patients compared to other groups, except for NA levels. NA is directly metabolized from QUIN in the KP by nicotinamide mononucleotide adenylyl transferase (NMNAT). Hence, it is expected that NA levels would decrease accordingly in ALS patients. However, as the study by Brazill et al. points out [59], NMNAT hyperactivity seems to have an neuroprotective effect in mice with natural NMNAT overexpression. This suggests that an increased conversion of QUIN to NA in ALS might also fit in the hypothesis of less kynurenine-related neuroinflammation in ALS patients. Following this analogy, we might also expect an increase in other neuroprotective compounds, such as KYNA in ALS patients, which was not the case in our study.

Allocation of patients to a diagnostic group was based on clinical diagnoses, but diagnosis was also neuropathologically confirmed postmortem. Hence, we hypothesized that we would observe within-group alterations in KP levels based on their neuropathological status; however, this was not the case. This could be a consequence of the relatively small subgroups within the diagnostic categories, as well as incomplete neuropathological information for some diagnostic groups (FTD–ALS and ALS).

As mentioned earlier, nearly all kynurenine compounds were consistently lower in ALS compared to FTD patients in all brain regions. Since both disease entities affect different brain structures, we might expect to see more neuroinflammation and KP activity in BA22 and the hippocampus (frontotemporal structures) in FTD patients, whereas we could expect higher neuroinflammation levels in ALS compared to FTD patients in motor-associated areas such as the neostriatum, substantia nigra and BA6. However, this was not observed in our study. This could signify that altered KP activity in FTD and ALS patients is not brain region specific, but rather a widespread phenomenon. On the other hand, neuroinflammation in ALS might be more concentrated in the spinal cord.

Throughout the analysis, ANA levels and KYN/TRP ratios were consistently lower in ALS compared to FTD and EOAD patients in all investigated brain regions. Hence, their levels may be useful as markers for neuroinflammation or to discriminate between ALS and FTD or EOAD patients. Evidently, this will require additional research into these metabolites and ratios in other matrices, such as CSF or blood, to reveal whether these differences between ALS and FTD or EOAD are comparable across the brain and biofluids. Eventually, this may result in the development of novel fluid biomarkers that could expand the panel of classic diagnostic criteria and biomarkers to further optimize diagnostic accuracy [60].

Interestingly, our findings are not entirely consistent with previous studies researching KP levels in ALS or FTD. For example, Chen et al. reported increased levels of CSF or serum KYN, TRP and QUIN in ALS compared to control individuals, whereas we observed significantly lower KYN and QUIN levels in BA22, BA6, BA10, the hippocampus and neostriatum. However, similar to our results, Chen and colleagues also described lower CSF and serum PA levels in ALS compared to control subjects. It should be mentioned, however, that there was a large difference in recruitment age in their study. The mean age of healthy control individuals was 35.8 ± 3.0 years compared to 58.3 ± 12.3 and 58.2 ± 12.0 years for female and male ALS patients, respectively [30]. In our study, ALS patients had the lowest median age. This could explain the large contrast with their results and might indeed point to an important aging effect on KP activity. In a study by Ilzecka et al., CSF KYNA levels were higher in patients with ALS with bulbar onset compared to control individuals or patients with limb onset. However, they did not observe this when comparing the whole group of ALS patients to control individuals. In addition, lower serum KYNA levels were observed in patients with a more severe clinical status as opposed to ALS patients with a mild clinical status or control individuals, which is consistent with our results. Ilzecka and colleagues did not find any correlation between age and KYNA levels [36]. It is also worth noting that the control group in their study did not consist of healthy subjects, but patients with acute vasomotor headaches [61], a condition linked to the serotonergic system as well [58]. This might also explain why there seems to be little consistency between both studies. In 2020, our research group reported no differences in KP compounds in CSF and the serum of FTD versus ALS patients, except for the serum 3-HK/XA ratio, which was significantly lower in ALS than in FTD patients [34]. This is in contrast with our findings; whereas there are consistent differences in all other kynurenine compounds, the 3-HK/XA ratio was unaltered between all diagnostic groups in all brain regions. The experimental setup was equal to the one used in our study. However, this discrepancy between CSF, serum and brain KP levels might be caused by varying abilities of kynurenine compounds to pass the blood–brain barrier and brain–CSF barrier, as mentioned earlier [45,46].

It is important to note that the KP is intimately connected with multiple neurotransmitters and hormonal systems across the brain, and alterations in one system may affect others and vice versa [62]. For example, TRP is not only a precursor to L-kynurenine and the first step in the kynurenine pathway, but it is also the substrate for the serotonergic metabolic pathway. In this way, both the kynurenine and the serotonergic pathway compete for TRP [62,63]. Moreover, the conversion of TRP to KYN can be promoted through the activation of TDO or IDO by the stress hormone cortisol. KYN can be either be converted to KYNA, a glutamatergic NMDA receptor agonist and considered neuroprotective, or it can be further converted to 3-HK and then to QUIN, a glutamatergic NMDA receptor antagonist considered neurotoxic in high levels [32]. In addition, KP components such as KYNA modulate the activity of the α7-nicotinic acetylcholine receptor (α7nAChR) and in this way the non-α7nAChR-mediated release of noradrenaline, dopamine and acetylcholine [62]. Considering the close link between these different neurochemical systems, we also investigated any potential association between KP levels and psychotropic medication use or other medication types that may directly or indirectly affect KP levels, such as corticosteroids and hormones. There were significant differences in medication use between all five diagnostic categories. As can be expected, antipsychotic and antidepression use was significantly higher in EOAD than in CONTR subjects. CNS-acting medication use was significantly higher in ALS patients than in all other patient groups, since all ALS patients for which medication use was recorded (five out of twenty) received treatment with the ALS drug riluzole. QUIN and riluzole are a glutamate NMDA receptor agonist and antagonist, respectively. Thus, although riluzole is a non-competitive antagonist, treatment with riluzole might affect the activity of QUIN. However, a regression analysis did not reveal any significant effect of CNS-acting medication on QUIN levels in the entire population. On the contrary, a significantly inverse relation with ANA levels was found, although this did not persist when only including patients taking CNS-acting medication (ALS and FTD/ALS). We did find a significant difference in BA6 QUIN levels for patients taking versus not taking psychotropic medication in general. However, when comparing KP levels in patients on psychotropic medication versus medication-free patients between all five groups, there were no differences that remained significant following post hoc correction. This could be a consequence of the small number of medication-free patients in some groups (e.g., there were no medication-free FTD–ALS patients because they all took at least one type of psychotropic medication).

There are some limitations to this study that need to be addressed. Firstly, the diagnostic groups were not age matched, which may underly the decreased KP levels in ALS patients compared to the other groups. Secondly, we did not have complete demographic information for every patient. For example, ALS subtype information was not available for all ALS patients in our study. As reported by Ilzecka et al., KP levels might differ between distinct ALS types [36]. In addition, there were a large number of patients for which medication information was not available. This makes it more difficult to assess the effect of (psychotropic) medication on the KP. Other potential confounding factors comprise the preanalytical steps. From sample preparation to long sample storage, these are all sources of variability that might affect KP levels. We did not investigate diet, although TRP is one of the essential amino acids derived from dietary uptake [64]. Most importantly, the statistical power was markedly reduced at times. Overall, although diagnostic groups were sufficiently large (from 11 to 25 subjects), not all kynurenine compounds could be reliably detected in every sample, which sometimes impacted the number of available samples per group, brain region and compound. Nevertheless, we were able to detect significant differences between groups and across brain regions, which points to a strong and consistent difference in KP activity.

## 4. Materials and Methods

### 4.1. Study Population

For this retrospective study, frozen brain samples from 98 subjects were selected or regionally dissected from the frozen left hemisphere according to a protocol described elsewhere [65]. The aforementioned samples originated from the NeuroBiobank of the Institute Born-Bunge (NBB-IBB (no. BB190113), Wilrijk, Belgium) and the Brain Biobanc Barcelona (Biobanc Hospital Clínic—IDIBAPS, Barcelona, Spain). All included patients were diagnosed with neuropathologically confirmed FTD (n = 24), ALS (n = 20), FTD–ALS (n = 11) or EOAD (n = 23). Neuropathological confirmation of AD-related pathology was based on ABC scoring, comprising Braak and Braak staging for neurofibrillary tangles, a four-phase scale Thal scoring for amyloid plaque deposition and CERAD scoring for neuritic plaque presence [66,67,68,69]. For FTD, ALS and FTD–ALS, the Brettschneider criteria and Mackenzie classification system were included to confirm the clinical diagnosis [57,70]. Healthy control (CONTR) subjects (n = 20) were included as described by Janssens et al. [34]. Patients with a history of psychiatric conditions or central nervous system pathology other than FTD, ALS or EOAD were excluded. Control subjects came to the hospital for lumbar canal stenosis, (tension) headaches, cervical myelopathy, carpal tunnel syndrome, chronic gait disorders, nausea, periodic fever syndrome, leucopenia, struma simplex, facial arteriovenous malformations and sinusitis.

### 4.2. Brain Dissections

The median post-mortem delay was approximately 4.5 h (interquartile range: 3h00–7h00). For brain samples from the NBB-IBB, a brain autopsy was followed by freezing of the left hemisphere at −80 °C for neurochemical analysis and fixation of the right hemisphere in paraformaldehyde (12%) for neuropathological examination. Regional brain dissection of the left frozen hemisphere was performed in accordance with the standard protocol during which 21 brain regions were dissected [71]. A selection of six neurochemically relevant brain regions, being Brodmann area (BA)6 (premotor cortex and supplementary motor area), BA10 (medial and prefrontal cortex), BA22 (posterior superior temporal cortex), substantia nigra, hippocampus and neostriatum (caudate nucleus and putamen), were analyzed by solid-phase extraction liquid chromatography coupled to tandem mass spectrometry (SPE-LC–MS/MS) to determine KP compounds.

### 4.3. LC-MS/MS

At the Department of Laboratory Medicine of the University Medical Center Groningen, online solid-phase extraction liquid chromatographic–tandem mass spectrometry with deuterated or ^13^C-labeled internal standards was applied to determine the concentrations of 3-hydroxykynurenine (3-HK), L-kynurenine (KYN), tryptophan (TRP), kynurenic acid (KYNA), quinolinic acid (QUIN), xanthurenic acid (XA), picolinic acid (PA), nicotinic acid (NA) and anthranilic acid (ANA) in the aforementioned brain regions. 

TRP, KYN and 3-HK were analyzed according to a previously described method [72]. KYNA, XA, PA, NA, ANA and QUIN were analyzed essentially as previously described [73]. In short, brain sample was homogenized in the presence of deuterated analogues ((^2^H_5_) KYNA, (^2^H_4_) XA, (^2^H_4_) PA and (^2^H_3_) QUIN) or ^13^C-labeled internal standards ((^13^C_6_) 3-HK, (^13^C_6_) KYN, (^13^C_11_) TRP, (^13^C_6_) NA and (^13^C_6_) ANA). In the next step, the mixtures were extracted using Phenomenex^®^ Strata X-A 96-well plates with a pore size of 33 µm and a sorbent mass of 30 mg/well (Cat. No.: 8E-S123-TGB; Strata-X, Phenomenex, Utrecht, The Netherlands) and eluted with 3 M HCl in 1-butanol. Following sample clean up, 1 µL was injected into a Waters^®^ Acquity UHPLC (Waters, Etten-Leur, The Netherlands) coupled to a Phenomenex^®^ Luna column (Omega C18, 100  ×  2.1 mm, particle size 1.6 µm, Phenomenex, Utrecht, The Netherlands) and to a XEVO TQ-S MS/MS system (Waters, Etten-Leur, The Netherlands). The interassay imprecision was determined by analyzing three different plasma pools at three different concentrations over n = 19 different days. Results are given in Appendix A, Table A6. The limit of quantification was calculated by analyzing the signal to noise ratio of different samples (n = 10) low in concentration and calculating the mean of the concentration at a signal to noise ratio of ≥ 10. 

Additionally, KYN/TRP and 3-HK/XA ratios were calculated as a measure for TDO or IDO activity and as an indicator of vitamin B6 function, respectively [74,75]. The latter determines 3-HK/XA ratios as kynurenine aminotransferase II, the enzyme that converts 3-HK to XA, is pyridoxal phosphate dependent [76,77].

### 4.4. Statistical Analysis

A Shapiro–Wilk normality test was performed to check the distribution of our study population. Following an abnormal distribution and the limited number of available samples in some of the diagnostic categories, non-parametric statistics were selected. Fisher’s exact tests were used to compare demographical parameters across groups, including male to female ratios and patients taking versus not taking psychotropic or central nervous system (CNS) medication. For age comparisons, as well as to detect differences in KP levels between the diagnostic categories, Kruskal–Wallis analyses (KW) with post hoc Dunn tests and adjusted Bonferroni correction were applied. The link between psychotropic medication use or sex with KP levels was investigated using Mann–Whitney U tests. In addition, Spearman correlation and regression analyses were performed to search for correlations between KP levels and age at death. Data were analyzed using SPSS 28 software for Windows (IBM, Armonk, NY, USA) and SigmaPlot 12.5 (Systat Software, Inc., San Jose, CA, USA).

## 5. Conclusions

This study offers exciting perspectives for future research. We set out to investigate commonalities and discrepancies in KP levels along the FTD–ALS continuum, and we were able to demonstrate robust differences between ALS, FTD, FTD–ALS, EOAD and control subjects. In particular, KP levels were consistently lower in ALS patients compared to FTD, EOAD and control individuals in all brain regions. Despite several limitations, this study provides a strong base for future researchers aiming to investigate KP alterations in early onset neurodegenerative disorders such as FTD, ALS and EOAD. One recommendation would be to use larger, age-matched populations who are medication-free or have full medication information available. It would be interesting to discover if this trend can be reproduced in future studies investigating KP metabolites in both brain regions and biofluids. A strong correspondence between brain, CSF and peripheral KP levels may propel the use of fluid kynurenine metabolites, such as ANA and the KYN/TRP ratio, as indicators for disease status or promising therapeutic targets.

## Figures and Tables

**Table 1:** Demographic and medication use. Age is depicted as the median with interquartile ranges between parentheses and was compared using Kruskal–Wallis with post hoc Dunn tests. Fisher’s Exact test was performed to compare the ratios of male to female subjects and patients not taking versus taking psychotropic medication. Statistics are listed in the rightmost column. Significant results following post hoc Dunn analysis with adjusted Bonferroni correction are classified as *p*-values *p* ≤ 0.05, *p* ≤ 0.01 and *p* ≤ 0.001 and are indicated by one, two or three superscript letters, respectively. The letters a, b, c and d denote significant differences between ALS and FTD^a^, ALS and EOAD^b^, ALS and CONTR^c^ and between EOAD and CONTR^d^. Abbreviations: ALS = amyotrophic lateral sclerosis, FTD = frontotemporal dementia, EOAD = early onset Alzheimer’s disease, CONTR = control individuals, CNS = central nervous system.

	ALS (n = 20)	FTD (n = 24)	FTD–ALS (n = 11)	EOAD (n = 23)	CONTR (n = 20)	Kruskal–Wallis
Age at death (years)	63.5 (54.2–67.5) ^c^	67.6 (60.9–73.4)	71.0 (64.7–76.1)	67.0 (59.5–72.2)	69.0 (62.8–78.0) ^c^	H = 10.777 *p* = 0.029
Sex (Male/Female)	6/14	14/10	6/5	14/9	12/8	Fisher’s Exact Test: 5.488 *p* > 0.05
Psychotropic medication (No/Yes/Missing)	0/5/15	1/17/6	2/2/7	2/20/1	7/7/6	Fisher’s Exact Test: 13.348 *p* = 0.004
Antidepressants (No/Yes/Missing)	1/4/15	9/9/6	4/0/7	10/12/1 ^ddd^	13/1/6 ^ddd^	Fisher’s Exact Test: 14.839 *p* = 0.003
Antipsychotics (No/Yes/Missing)	5/0/15	10/8/6	3/1/7	10/12/1 ^ddd^	13/1/6 ^ddd^	Fisher’s Exact Test: 11.927 *p* = 0.012
Antiepileptics (No/Yes/Missing)	4/1/15	15/3/6	4/0/7	21/1/1	14/0/6	Fisher’s Exact Test: 4.362 *p* > 0.05
Anxiolytics & hypnotics (No/Yes/Missing)	5/0/15	11/7/6	4/0/7	16/6/1	10/4/6	Fisher’s Exact Test: 3.783 *p* > 0.05
Analgesics (No/Yes/Missing)	3/2/15	12/6/6	4/0/7	17/5/1	10/4/6	Fisher’s Exact Test: 2.339 *p* > 0.05
Anticholinergic medication (No/Yes/Missing)	5/0/15	15/3/6	4/0/7	16/6/1	14/0/6	Fisher’s Exact Test: 5.501 *p* > 0.05
Antidementia medication (No/Yes/Missing)	5/0/15	14/4/6	4/0/7	13/9/1	14/0/6	Fisher’s Exact Test: 10.085 *p* = 0.023
CNS-acting medication (No/Yes/Missing)	0/5/15 ^aaa,bbb,ccc^	18/0/6 ^aaa^	3/1/7	22/0/1 ^bbb^	14/0/6 ^ccc^	Fisher’s Exact Test: 28.277 *p* < 0.001
Hormones (No/Yes/Missing)	5/0/15	17/1/6	4/0/7	21/1/1	13/1/6	Fisher’s Exact Test: 1.486 *p* > 0.05
Corticosteroids (No/Yes/Missing)	5/0/15	17/1/6	4/0/7	22/0/1	13/1/6	Fisher’s Exact Test: 3.200 *p* > 0.05
Adrenergic medication (No/Yes/Missing)	4/1/15	18/0/6	4/0/7	22/0/1	11/3/6	Fisher’s Exact Test: 8.047 *p* = 0.029
Antiemetics (No/Yes/Missing)	4/1/15	18/0/6	4/0/7	21/1/1	13/1/6	Fisher’s Exact Test: 4.408 *p* > 0.05
Antiparkinson medication (No/Yes/Missing)	5/0/15	16/2/6	4/0/7	21/1/1	14/0/6	Fisher’s Exact Test: 2.484 *p* > 0.05

**Table 2:** Brodmann area 22 kynurenine pathway metabolite levels. Data are represented as median with interquartile ranges between brackets. Kruskal–Wallis with post hoc Dunn tests were performed to compare levels of kynurenine pathway components between five diagnostic categories. Statistical results are listed in the two rightmost columns. Psychotropic medication free groups consisted of no ALS, one FTD, two FTD–ALS, two EOAD patients and seven CONTR individuals. Adjusted significance values are classified as *p* ≤ 0.05, *p* ≤ 0.01 and *p* ≤ 0.001 and are indicated by one, two or three superscript letters, respectively. The letters a, b, c and d denote significant differences between ALS and FTD^a^, ALS and EOAD^b^_,_ ALS and CONTR^c^ and between FTD and FTD–ALS^d^. Abbreviations: ALS = amyotrophic lateral sclerosis, FTD = frontotemporal dementia, EOAD = early onset Alzheimer’s disease, CONTR = control individuals, 3-HK = 3-hydroxykynurenine, KYN = kynurenine, TRP = tryptophan, KYNA = kynurenic acid, XA = xanthurenic acid, QUIN = quinolinic acid, PA = picolinic acid, NA = nicotinic acid, ANA = anthranilic acid.

	ALS (n = 20)	FTD (n = 24)	FTD–ALS (n = 11)	EOAD (n = 23)	CONTR (n = 20)	Kruskal–Wallis	Kruskal–Wallis (Psychotropic Medication Free)
3-HK (pmol/mg)	0.179 (0.130–0.240) (n = 20) ^aa^	0.382 (0.284–0.646) (n = 23) ^aa,d^	0.129 (0.072–0.413) (n = 11) ^d^	0.243 (0.114–0.457) (n = 14)	0.315 (0.167–1.466) (n = 7)	H = 21.832 *p* < 0.001	H = 4.250 *p* > 0.05
KYN (pmol/mg)	1.119 (0.660–1.655) (n = 20) ^aa,b^	2.808 (1.781–4.865) (n = 23) ^aa^	1.069 (0.887–2.957) (n = 11)	2.680 (1.775–5.164) (n = 14) ^b^	4.467 (0.670–5.621) (n = 7)	H = 18.205 *p* = 0.001	H = 3.583 *p* > 0.05
TRP (pmol/mg)	49.790 (43.674–61.748) (n =20)	63.595 (44.317–80.268) (n = 23)	52.714 (31.859–76.818) (n = 11)	64.399 (43.471–80.521) (n = 14)	57.480 (35.792–68.562) (n = 7)	H = 6.340 *p* > 0.05	H = 0.500 *p* > 0.05
KYNA (pmol/mg)	0.247 (0.186–0.405) (n = 20)	0.405 (0.200–0.859) (n = 23)	0.365 (0.157–0.622) (n = 11)	0.376 (0.301–0.723) (n = 14)	0.575 (0.134–1.480) (n = 7)	H = 6.902 *p* > 0.05	H = 2.056 *p* > 0.05
XA (pmol/mg)	0.003 (0.002–0.004) (n = 20)	0.007 (0.003–0.014) (n = 23)	0.003 (0.002–0.006) (n = 11)	0.008 (0.003–0.013) (n = 14)	0.009 (0.003–0.022) (n = 7)	H = 18.207 *p* = 0.001	H = 5.389 *p* > 0.05
QUIN (pmol/mg)	0.031 (0.020–0.076) (n = 20) ^aa^	0.115 (0.079–0.289) (n = 23) ^aa^	0.109 (0.032–0.210) (n = 11)	0.144 (0.046–0.291) (n = 14)	0.281 (0.037–0.636) (n = 7)	H = 18.865 *p* < 0.001	H = 4.722 *p* > 0.05
PA (pmol/mg)	0.036 (0.024–0.096) (n = 20) ^aa^	0.126 (0.039–0.243) (n = 23) ^aa^	0.063 (0.030–0.205) (n = 11)	0.122 (0.068–0.374) (n = 14)	0.145 (0.038–0.217) (n = 7)	H = 14.833 *p* = 0.005	H = 5.139 *p* > 0.05
NA (pmol/mg)	9.879 (7.085–12.940) (n = 20) ^b^	8.178 (5.613–12.078) (n = 23)	7.564 (4.855–10.562) (n = 11)	5.642 (3.810–7.730) (n = 14) ^b^	8.964 (4.119–10.348) (n = 7)	H = 10.965 *p* = 0.030	H = 4.806 *p* > 0.05
ANA (pmol/mg)	0.043 (0.016–0.065) (n = 20) ^aaa,bbb,cc^	0.115 (0.082–0.181) (n = 23) ^aaa^	0.087 (0.033–0.137) (n = 11)	0.171 (0.084–0.305) (n = 14) ^bbb^	0.144 (0.107–0.193) (n = 7) ^cc^	H = 27.681 *p* < 0.001	H = 6.500 *p* > 0.05
KYN/TRP	0.023 (0.013–0.031) (n = 20) ^aa^	0.041 (0.028–0.053) (n = 23) ^aa^	0.034 (0.019–0.044) (n = 11)	0.033 (0.023–0.059) (n = 14)	0.052 (0.017–0.082) (n = 7)	H = 14.879 *p* = 0.005	H = 3.583 *p* > 0.05
3-HK/XA	64.377 (47.660–91.572) (n = 20)	62.705 (43.536–92.817) (n = 23)	64.260 (37.645–83.200) (n = 11)	36.403 (21.814–63.653) (n = 14)	64.857 (41.607–77.034) (n = 7)	H = 7.918 *p* > 0.05	H = 2.333 *p* > 0.05

## Data Availability

The data that support the findings of this study are available from the corresponding author upon reasonable request.

## Appendix A

**Table A1:** Brodmann area 6 kynurenine pathway metabolite levels.

	ALS (n = 20)	FTD (n = 24)	FTD–ALS (n = 11)	EOAD (n = 23)	CONTR (n = 20)	Kruskal–Wallis	Kruskal–Wallis (Psychotropic Medication Free)
3-HK (pmol/mg)	0.097 (0.068–0.149) (n =5) ^c^	0.271 (0.187–0.432) (n = 23)	0.100 (0.059–0.369) (n = 4)	0.230 (0.121–0.363) (n = 20)	0.277 (0.151–1.176) (n = 16) ^c^	H = 12.073 *p* = 0.017	H = 4.319 *p* > 0.05
KYN (pmol/mg)	0.826 (0.519–1.227) (n = 5)	2.691 (1.768–3.882) (n = 23)	0.874 (0.595–3.434) (n = 4)	2.793 (1.814–4.488) (n = 20)	1.901 (0.816–3.467) (n = 16)	H = 3.673 *p* = 0.042	H = 5.819 *p* > 0.05
TRP (pmol/mg)	50.684 (36.098–55.231) (n =5)	59.745 (38.969–89.684) (n = 23)	41.376 (31.418–62.697) (n = 4)	59.895 (37.411–75.573) (n = 20)	47.572 (41.129–62.970) (n = 16)	H = 4.309 *p* > 0.05	H = 2.060 *p* > 0.05
KYNA (pmol/mg)	0.197 (0.163–0.275) (n = 5)	0.471 (0.279–0.670) (n = 23)	0.272 (0.105–0.953) (n = 4)	0.608 (0.313–0.967) (n = 20)	0.584 (0.251–1.108) (n = 16)	H = 8.938 *p* > 0.05	H = 5.736 *p* > 0.05
XA (pmol/mg)	0.002 (0.001–0.002) (n = 5)	0.006 (0.002–0.011) (n = 23)	0.006 (0.002–0.017) (n = 4)	0.010 (0.004–0.015) (n = 20)	0.010 (0.003–0.027) (n = 16)	H = 9.459 *p* > 0.05	H = 3.934 *p* > 0.05
QUIN (pmol/mg)	0.019 (0.003–0.043) (n = 5) ^c^	0.090 (0.062–0.193) (n = 23)	0.021 (0.006–1.050) (n = 4)	0.087 (0.041–0.221) (n = 20)	0.195 (0.040–0.587) (n = 16) ^c^	H = 10.879 *p* = 0.028	H = 6.176 *p* > 0.05
PA (pmol/mg)	0.019 (0.018–0.070) (n = 5)	0.120 (0.036–0.240) (n = 23)	0.049 (0.025–0.192) (n = 4)	0.150 (0.046–0.357) (n = 20)	0.123 (0.045–0.242) (n = 16)	H = 8.056 *p* > 0.05	H = 6.280 *p* > 0.05
NA (pmol/mg)	4.322 (3.201–7.958) (n = 5)	8.533 (4.509–10.374) (n = 23)	9.128 (4.908–12.643) (n = 4)	8.075 (5.557–11.014) (n = 20)	8.109 (6.240–9.677) (n = 16)	H = 4.363 *p* > 0.05	H = 1.077 *p* > 0.05
ANA (pmol/mg)	0.014 (0.003–0.029) (n = 5) ^a,bbb^	0.088 (0.060–0.131) (n = 23) ^a^	0.057 (0.018–0.122) (n = 4)	0.136 (0.083–0.260) (n = 20) ^bbb^	0.069 (0.035–0.157) (n = 16)	H = 19.963 *p* < 0.001	H = 6.396 *p* > 0.05
KYN/TRP	0.015 (0.014–0.025) (n = 5) ^a,b^	0.041 (0.028–0.055) (n = 23) ^a^	0.028 (0.014–0.056) (n = 4)	0.039 (0.029–0.063) (n = 20) ^b^	0.0365 (0.025–0.070) (n = 16)	H = 10.961 *p* = 0.027	H = 4.670 *p* > 0.05
3-HK/XA	82.000 (45.626–83.693) (n = 5)	56.810 (37.367–87.727) (n = 23)	34.670 (9.524–46.561) (n = 4)	30.762 (15.469–57.833) (n = 20)	45.744 (28.636–80.060) (n = 16)	H = 7.916 *p* > 0.05	H = 5.308 *p* > 0.05

Data are represented as medians with interquartile ranges between brackets. Kruskal–Wallis with post hoc Dunn tests were performed to compare levels of kynurenine pathway components between five diagnostic categories. Statistical results are listed in the two rightmost columns. Psychotropic-medication-free groups consisted of zero ALS, one FTD, two FTD–ALS, two EOAD patients and seven CONTR individuals. Adjusted significance values are classified as *p* ≤ 0.05 and *p* ≤ 0.001 and are indicated by one or three superscript letters, respectively. The letters a, b and c denote significant differences between ALS and FTD^a^, ALS and EOAD^b^ and ALS and CONTR^c^. Abbreviations: ALS = amyotrophic lateral sclerosis, FTD = frontotemporal dementia, EOAD = early onset Alzheimer’s disease, CONTR = control individuals, 3-HK = 3-hydroxykynurenine, KYN = kynurenine, TRP = tryptophan, KYNA = kynurenic acid, XA = xanthurenic acid, QUIN = quinolinic acid, PA = picolinic acid, NA = nicotinic acid, ANA = anthranilic acid.

**Table A2:** Brodmann area 10 kynurenine pathway metabolite levels.

	ALS (n = 20)	FTD (n = 24)	FTD–ALS (n = 11)	EOAD (n = 23)	CONTR (n = 20)	Kruskal–Wallis	Kruskal–Wallis (Psychotropic Medication Free)
3-HK (pmol/mg)	0.149 (0.089–0.181) (n =20) ^aa,ccc^	0.316 (0.211–0.470) (n = 23) ^aa^	0.174 (0.084–0.297) (n = 11) ^d^	0.244 (0.154–0.428) (n = 23)	0.547 (0.227–1.353) (n = 19) ^ccc,d^	H = 26.169 *p* < 0.001	H = 4.368 *p* > 0.05
KYN (pmol/mg)	1.065 (0.657–1.381) (n = 20) ^aa,bb,c^	2.399 (1.894–3.574) (n = 23) ^aa^	1.442 (0.760–8.984) (n = 11)	2.569 (1.714–3.774) (n = 23) ^bb^	2.500 (1.130–3.510) (n = 19) ^c^	H = 19.692 *p* < 0.001	H = 5.819 *p* > 0.05
TRP (pmol/mg)	43.599 (37.866–49.496) (n =20)	58.552 (39.385–76.132) (n = 23)	40.469 (33.934–60.961) (n = 11)	50.185 (37.389–65.420) (n = 23)	51.375 (42.655–60.816) (n = 19)	H = 7.567 *p* > 0.05	H = 4.280 *p* > 0.05
KYNA (pmol/mg)	0.223 (0.152–0.368) (n = 20)	0.285 (0.171–0.390) (n = 23)	0.292 (0.105–0.701) (n = 11)	0.398 (0.206–0.529) (n = 23)	0.435 (0.190–0.564) (n = 19)	H = 4.791 *p* > 0.05	H = 5.357 *p* > 0.05
XA (pmol/mg)	0.002 (0.001–0.003) (n = 20) ^bbb,ccc^	0.005 (0.003–0.009) (n = 23)	0.004 (0.002–0.005) (n = 11)	0.007 (0.003–0.013) (n = 23) ^bbb^	0.008 (0.003–0.027) (n = 19) ^ccc^	H = 20.455 *p* < 0.001	H = 5.165 *p* > 0.05
QUIN (pmol/mg)	0.025 (0.017–0.047) (n = 20) ^a,b,ccc^	0.101 (0.059–0.189) (n = 23) ^a^	0.088 (0.036–0.219) (n = 11)	0.080 (0.034–0.214) (n = 23) ^b^	0.226 (0.050–0.653) (n = 19) ^ccc^	H = 23.314 *p* < 0.001	H = 6.176 *p* > 0.05
PA (pmol/mg)	0.031 (0.019–0.082) (n = 20) ^a,bb,c^	0.118 (0.033–0.227) (n = 23) ^a^	0.066 (0.033–0.132) (n = 11)	0.128 (0.045–0.394) (n = 23) ^bb^	0.157 (0.066–0.220) (n = 19) ^c^	H = 15.938 *p* = 0.003	H = 6.429 *p* > 0.05
NA (pmol/mg)	9.736 (6.906–10.929) (n = 20) ^aaa,b^	5.147 (3.503–6.601) (n = 23) ^aaa^	6.383 (4.466–7.816) (n = 11)	6.321 (4.858–7.336) (n = 23) ^b^	6.615 (5.533–7.477) (n = 19)	H = 22.059 *p* < 0.001	H = 5.560 *p* > 0.05
ANA (pmol/mg)	0.033 (0.010–0.060) (n = 20) ^aa,bbb,c^	0.089 (0.057–0.132) (n = 23) ^aa^	0.084 (0.029–0.125) (n = 11)	0.139 (0.080–0.196) (n = 23) ^bbb^	0.068 (0.043–0.134) (n = 19) ^c^	H = 30.018 *p* < 0.001	H = 6.636 *p* > 0.05
KYN/TRP	0.023 (0.015–0.032) (n = 20) ^aa,bbb,c^	0.043 (0.028–0.053) (n = 23) ^aa^	0.039 (0.021–0.047) (n = 11)	0.042 (0.036–0.074) (n = 23) ^bbb^	0.043 (0.023–0.071) (n = 19) ^c^	H = 20.528 *p* < 0.001	H = 5.088 *p* > 0.05
3-HK/XA	63.232 (44.304–78.804) (n = 20)	68.138 (40.067–101.081) (n = 23)	59.820 (23.694–65.210) (n = 11)	38.242 (16.459–62.043) (n = 23)	59.362 (33.222–102.185) (n = 19)	H = 8.826 *p* > 0.05	H = 3.511 *p* > 0.05

Data are represented as median with interquartile ranges between brackets. Kruskal–Wallis with post hoc Dunn tests were performed to compare levels of kynurenine pathway components between five diagnostic categories. Statistical results are listed in the two rightmost columns. Psychotropic-medication-free groups consisted of zero ALS, one FTD, two FTD–ALS, two EOAD patients and seven CONTR individuals. Adjusted significance values are classified as *p* ≤ 0.05, *p* ≤ 0.01 and *p* ≤ 0.001 and are indicated by one, two or three superscript letters, respectively. The letters a, b, c and d denote significant differences between ALS and FTD^a^, ALS and EOAD^b^_,_ ALS and CONTR^c^ and between FTD–ALS and CONTR^d^. Abbreviations: ALS = amyotrophic lateral sclerosis, FTD = frontotemporal dementia, EOAD = early onset Alzheimer’s disease, CONTR = control individuals, 3-HK = 3-hydroxykynurenine, KYN = kynurenine, TRP = tryptophan, KYNA = kynurenic acid, XA = xanthurenic acid, QUIN = quinolinic acid, PA = picolinic acid, NA = nicotinic acid, ANA = anthranilic acid.

**Table A3:** Substantia nigra kynurenine pathway metabolite levels.

	ALS (n = 20)	FTD (n = 24)	FTD–ALS (n = 11)	EOAD (n = 23)	CONTR (n = 20)	Kruskal–Wallis	Kruskal–Wallis (Psychotropic Medication Free)
3-HK (pmol/mg)	0.045 (0.035–0.152) (n =5)	0.171 (0.090–0.266) (n = 21)	0.047 (0.033–0.445) (n = 4)	0.154 (0.060–0.223) (n = 22)	0.221 (0.101–1.018) (n = 15)	H = 9.109 *p* > 0.05	H = 5.617 *p* > 0.05
KYN (pmol/mg)	0.960 (0.844–1.560) (n = 5)	2.563 (1.885–4.069) (n = 21)	1.208 (0.792–3.464) (n = 4)	2.708 (1.848–3.738) (n = 22)	2.601 (1.251–3.541) (n = 15)	H = 6.280 *p* > 0.05	H = 3.461 *p* > 0.05
TRP (pmol/mg)	72.913 (54.697–77.153) (n = 5)	66.919 (42.826–87.649) (n = 21)	78.706 (42.791–108.929) (n = 4)	62.102 (42.911–90.552) (n = 22)	68.146 (57.883–95.652) (n = 15)	H = 1.812 *p* > 0.05	H = 4.604 *p* > 0.05
KYNA (pmol/mg)	0.221 (0.198–0.365) (n = 5)	0.463 (0.299–0.642) (n = 21)	0.328 (0.197–0.860) (n = 4)	0.479 (0.251–0.823) (n = 22)	0.611 (0.256–0.783) (n = 15)	H = 5.113 *p* > 0.05	H = 5.695 *p* > 0.05
XA (pmol/mg)	0.001 (0.000–0.002) (n = 5)	0.005 (0.002–0.008) (n = 21)	0.002 (0.000–0.019) (n = 4)	0.008 (0.002–0.016) (n = 22)	0.005 (0.001–0.017) (n = 15)	H = 7.521 *p* > 0.05	H = 4.431 *p* > 0.05
QUIN (pmol/mg)	0.020 (0.003–0.0145) (n = 5)	0.109 (0.073–0.227) (n = 21)	0.033 (0.008–1.315) (n = 4)	0.127 (0.028–0.239) (n = 22)	0.220 (0.059–0.848) (n = 15)	H = 7.892 *p* > 0.05	H = 5.485 *p* > 0.05
PA (pmol/mg)	0.041 (0.022–0.086) (n = 5)	0.096 (0.038–0.245) (n = 21)	0.067 (0.026–0.173) (n = 4)	0.114 (0.046–0.415) (n = 22)	0.120 (0.041–0.241) (n = 15)	H = 4.432 *p* > 0.05	H = 6.649 *p* > 0.05
NA (pmol/mg)	6.018 (4.471–10.923) (n = 5)	5.565 (4.155–6.690) (n = 21)	6.495 (4.072–10.224) (n = 4)	7.504 (5.426–8.194) (n = 22)	6.843 (4.806–9.882) (n = 15)	H = 6.104 *p* > 0.05	H = 1.071 *p* > 0.05
ANA (pmol/mg)	0.023 (0.000–0.043) (n = 5) ^bb^	0.116 (0.069–0.136) (n = 21)	0.074 (0.009–0.151) (n = 4)	0.162 (0.083–0.221) (n = 22) ^bb^	0.059 (0.040–0.156) (n = 15)	H = 16.271 *p* = 0.003	H = 6.162 *p* > 0.05
KYN/TRP	0.014 (0.012–0.027) (n = 5) ^b^	0.039 (0.027–0.050) (n = 21)	0.024 (0.011–0.038) (n = 4)	0.036 (0.026–0.052) (n = 22) ^b^	0.027 (0.018–0.056) (n = 15)	H = 10.658 *p* = 0.031	H = 5.325 *p* > 0.05
3-HK/XA	106.530 (35.265–295.489) (n = 5)	45.478 (16.334–81.452) (n = 21)	36.504 (12.886–1107.970) (n = 4)	21.733 (10.640–59.994) (n = 22)	49.111 (26.341–174.329) (n = 15)	H = 8.443 *p* > 0.05	H = 0.779 *p* > 0.05

Data are represented as median with interquartile ranges between brackets. Kruskal–Wallis with post hoc Dunn tests were performed to compare levels of kynurenine pathway components between five diagnostic categories. Statistical results are listed in the two rightmost columns. Psychotropic-medication-free groups consisted of zero ALS, one FTD, two FTD–ALS, two EOAD patients and seven CONTR individuals. Adjusted significance values are classified as *p* ≤ 0.05 and *p* ≤ 0.01 and are indicated by one or two superscript letters, respectively. The letter b denotes significant differences between ALS and EOAD^b^. Abbreviations: ALS = amyotrophic lateral sclerosis, FTD = frontotemporal dementia, EOAD = early onset Alzheimer’s disease, CONTR = control individuals, 3-HK = 3-hydroxykynurenine, KYN = kynurenine, TRP = tryptophan, KYNA = kynurenic acid, XA = xanthurenic acid, QUIN = quinolinic acid, PA = picolinic acid, NA = nicotinic acid, ANA = anthranilic acid.

**Table A4:** Hippocampal kynurenine pathway metabolite levels.

	ALS (n = 20)	FTD (n = 24)	FTD–ALS (n = 11)	EOAD (n = 23)	CONTR (n = 20)	Kruskal–Wallis	Kruskal–Wallis (Psychotropic Medication Free)
3-HK (pmol/mg)	0.078 (0.051–0.106) (n =19) ^aaa,bb,ccc^	0.218 (0.152–0.384) (n = 22) ^aaa^	0.121 (0.065–0.213) (n = 11)	0.204 (0.087–0.336) (n = 22) ^bb^	0.280 (0.129–1.093) (n = 15) ^ccc^	H = 27.456 *p* < 0.001	H = 4.436 *p* > 0.05
KYN (pmol/mg)	1.041 (0.800–1.528) (n = 19) ^aa,bb,c^	2.691 (1.986–4.224) (n = 22) ^aa^	1.654 (0.772–3.037) (n = 11)	2.991 (1.820–4.493) (n = 22) ^bb^	2.554 (1.375–3.992) (n = 15) ^c^	H = 18.775 *p* < 0.001	H = 5.527 *p* > 0.05
TRP (pmol/mg)	70.418 (63.628–80.470) (n =19)	77.954 (47.530–94.132) (n = 22)	70.300 (40.110–94.641) (n = 11)	67.003 (38.662–90.356) (n = 22)	70.578 (61.559–90.414) (n = 15)	H = 1.444 *p* > 0.05	H = 0.182 *p* > 0.05
KYNA (pmol/mg)	0.129 (0.102–0.193) (n = 19) ^b^	0.233 (0.137–0.376) (n = 22)	0.225 (0.092–0.338) (n = 11)	0.332 (0.155–0.467) (n = 22) ^b^	0.235 (0.136–0.575) (n = 15)	H = 12.793 *p* = 0.012	H = 5.527 *p* > 0.05
XA (pmol/mg)	0.002 (0.001–0.003) (n = 19) ^a,bb,c^	0.005 (0.002–0.008) (n = 22) ^a^	0.003 (0.002–0.006) (n = 11)	0.008 (0.002–0.122) (n = 22) ^bb^	0.007 (0.002–0.018) (n = 15) ^c^	H = 16.989 *p* = 0.002	H = 3.732 *p* > 0.05
QUIN (pmol/mg)	0.026 (0.021–0.058) (n = 19) ^a,b,ccc^	0.091 (0.061–0.289) (n = 22) ^a^	0.078 (0.025–0.158) (n = 11)	0.126 (0.036–0.211) (n = 22) ^b^	0.251 (0.060–0.736) (n = 15) ^ccc^	H = 18.680 *p* < 0.001	H = 4.683 *p* > 0.05
PA (pmol/mg)	0.027 (0.019–0.092) (n = 19) ^a,b,c^	0.136 (0.040–0.258) (n = 22) ^a^	0.074 (0.044–0.150) (n = 11)	0.125 (0.045–0.378) (n = 22) ^b^	0.160 (0.047–0.210) (n = 15) ^c^	H = 14.362 *p* = 0.006	H = 4.655 *p* > 0.05
NA (pmol/mg)	11.287 (7.588–15.388) (n = 19) ^b^	7.096 (6.143–10.102) (n = 22)	8.932 (4.507–11.749) (n = 11)	6.661 (3.330–9.263) (n = 22) ^b^	8.528 (5.334–9.702) (n = 15)	H = 10.292 *p* = 0.036	H = 0.000 *p* > 0.05
ANA (pmol/mg)	0.041 (0.019–0.061) (n = 19) ^aa,bbb^	0.091 (0.051–0.144) (n = 22) ^aa^	0.098 (0.025–0.139) (n = 11)	0.093 (0.070–0.195) (n = 22) ^bbb^	0.063 (0.051–0.120) (n = 15)	H = 19.546 *p* < 0.001	H = 6.182 *p* = 0.045
KYN/TRP	0.018 (0.010–0.029) (n = 19) ^aa,bbb,c^	0.038 (0.024–0.050) (n = 22) ^aa^	0.032 (0.019–0.043) (n = 11)	0.042 (0.025–0.057) (n = 22) ^bbb^	0.038 (0.019–0.060) (n = 15) ^c^	H = 21.702 *p* < 0.001	H = 3.915 *p* > 0.05
3-HK/XA	56.835 (27.000–92.577) (n = 19)	55.206 (34.042–87.430) (n = 22)	38.211 (24.360–70.876) (n = 11)	30.668 (11.502–67.397) (n = 22)	61.354 (33.220–154.368) (n = 15)	H = 7.698 *p* > 0.05	H = 0.295 *p* > 0.05

Data are represented as median with interquartile ranges between brackets. Kruskal–Wallis with post hoc Dunn tests were performed to compare levels of kynurenine pathway components between five diagnostic categories. Statistical results are listed in the two rightmost columns. Psychotropic-medication-free groups consisted of zero ALS, one FTD, two FTD–ALS, two EOAD patients and seven CONTR individuals. Adjusted significance values are classified as *p* ≤ 0.05, *p* ≤ 0.01 and *p* ≤ 0.001 and are indicated by one, two or three superscript letters, respectively. The letters a, b and c denote significant differences between ALS and FTD^a^, ALS and EOAD^b^ and ALS and CONTR^c^. Abbreviations: ALS = amyotrophic lateral sclerosis, FTD = frontotemporal dementia, EOAD = early onset Alzheimer’s disease, CONTR = control individuals, 3-HK = 3-hydroxykynurenine, KYN = kynurenine, TRP = tryptophan, KYNA = kynurenic acid, XA = xanthurenic acid, QUIN = quinolinic acid, PA = picolinic acid, NA = nicotinic acid, ANA = anthranilic acid.

**Table A5:** Neostriatal kynurenine pathway metabolite levels.

	ALS (n = 20)	FTD (n = 24)	FTD–ALS (n = 11)	EOAD (n = 23)	CONTR (n = 20)	Kruskal–Wallis	Kruskal–Wallis (Psychotropic Medication Free)
3-HK (pmol/mg)	0.056 (0.042–0.095) (n = 19) ^aaa,bbb,ccc^	0.203 (0.130–0.363) (n = 24) ^aaa^	0.100 (0.047–0.192) (n = 11)	0.207 (0.088–0.298) (n = 23) ^bbb^	0.218 (0.099–0.762) (n = 20) ^ccc^	H = 28.811 *p* < 0.001	H = 5.736 *p* > 0.05
KYN (pmol/mg)	1.108 (0.853–1.696) (n = 19) ^aaa,bbb,cc^	3.141 (2.364–4.862) (n = 24) ^aaa^	2.545 (0.900–3.353) (n = 11)	3.989 (2.239–4.775) (n = 23) ^bbb^	3.564 (1.344–4.817) (n = 20) ^cc^	H = 25.847 *p* < 0.001	H = 6.615 *p* > 0.05
TRP (pmol/mg)	68.277 (48.502–82.532) (n = 19)	77.566 (53.803–109.793) (n = 24)	59.902 (42.723–73.137) (n = 11)	67.920 (48.997–104.054) (n = 23)	70.770 (61.050–84.172) (n = 20)	H = 6.231 *p* > 0.05	H = 4.236 *p* > 0.05
KYNA (pmol/mg)	0.280 (0.236–0.533) (n = 19) ^aa,bbb,cc^	0.781 (0.488–1.096) (n = 24) ^aa^	0.600 (0.282–0.988) (n= 11)	0.926 (0.424–1.282) (n = 23) ^bbb^	0.916 (0.339–1.342) (n = 20) ^cc^	H = 19.868 *p* < 0.001	H = 5.885 *p* > 0.05
XA (pmol/mg)	0.002 (0.001–0.003) (n = 19) ^aa,bbb,cc^	0.007 (0.002–0.013) (n = 24) ^aa^	0.003 (0.002–0.005) (n = 11)	0.010 (0.004–0.020) (n = 23) ^bbb^	0.007 (0.002–0.022) (n = 20) ^cc^	H = 21.450 *p* < 0.001	H = 5.078 *p* > 0.05
QUIN (pmol/mg)	0.042 (0.029–0.072) (n = 19) ^aaa,b,ccc^	0.142 (0.094–0.321) (n = 24) ^aaa^	0.096 (0.030–0.237) (n = 11)	0.161 (0.059–0.301) (n = 23) ^b^	0.262 (0.056–0.905) (n = 20) ^ccc^	H = 22.167 *p* < 0.001	H = 6.176 *p* > 0.05
PA (pmol/mg)	0.037 (0.021–0.090) (n = 19) ^a,b^	0.102 (0.049–0.276) (n = 24) ^a^	0.102 (0.048–0.163) (n = 11)	0.117 (0.047–0.435) (n = 23) ^b^	0.149 (0.056–0.220) (n = 20)	H = 13.247 *p* = 0.010	H = 6.265 *p* > 0.05
NA (pmol/mg)	11.759 (9.617–15.757) (n = 19) ^a^	8.743 (5.695–10.223) (n = 24) ^a^	10.043 (5.028–11.347) (n = 11)	10.708 (8.047–12.392) (n = 23)	10.264 (6.596–12.884) (n = 20)	H = 10.685 *p* = 0.030	H = 3.665 *p* > 0.05
ANA (pmol/mg)	0.046 (0.029–0.065) (n = 19) ^aa,bbb^	0.105 (0.060–0.129) (n = 24) ^aa^	0.107 (0.026–0.139) (n = 11)	0.111 (0.080–0.197) (n = 23) ^bbb^	0.066 (0.051–0.146) (n = 20)	H = 18.811 *p* < 0.001	H = 7.005 *p* > 0.05
KYN/TRP	0.020 (0.013–0.031) (n = 19) ^aa,bbb,c^	0.041 (0.027–0.061) (n = 24) ^aa^	0.034 (0.021–0.056) (n = 11)	0.046 (0.034–0.076) (n = 23) ^bbb^	0.041 (0.020–0.063) (n = 20) ^c^	H = 19.857 *p* < 0.001	H = 5.621 *p* > 0.05
3-HK/XA	36.663 (23.324–64.789) (n = 19)	37.106 (20.606–75.004) (n = 24)	35.959 (15.867–45.849) (n = 11)	22.208 (11.574–38.688) (n = 23)	37.123 (25.112–57.850) (n = 20)	H = 9.064 *p* > 0.05	H = 2.473 *p* > 0.05

Data are represented as median with interquartile ranges between brackets. Kruskal–Wallis with post hoc Dunn tests were performed to compare levels of kynurenine pathway components between five diagnostic categories. Statistical results are listed in the two rightmost columns. Psychotropic-medication-free groups consisted of zero ALS, one FTD, two FTD–ALS, two EOAD patients and seven CONTR individuals. Adjusted significance values are classified as *p* ≤ 0.05, *p* ≤ 0.01 and *p* ≤ 0.001 and are indicated by one, two or three superscript letters, respectively. The letters a, b and c denote significant differences between ALS and FTD^a^, ALS and EOAD^b^ and ALS and CONTR^c^. Abbreviations: ALS = amyotrophic lateral sclerosis, FTD = frontotemporal dementia, EOAD = early onset Alzheimer’s disease, CONTR = control individuals, 3-HK = 3-hydroxykynurenine, KYN = kynurenine, TRP = tryptophan, KYNA = kynurenic acid, XA = xanthurenic acid, QUIN = quinolinic acid, PA = picolinic acid, NA = nicotinic acid, ANA = anthranilic acid.

**Table A6:** Interassay imprecision (n = 19 different days) and LOQ.

		QC Low	QC Medium	QC High	LOQ
3-HK	Average	37.2	147	487	1.6
(µmol/L)	CV	5.8	4.4	3.7	
KYN	Average	1.79	4.6	17.3	0.0036
(nmol/L)	CV	4.7	4.1	3.0	
TRP	Average	52.5	109	271	0.17
(µmol/L)	CV	2.1	2.3	2.4	
KYNA	Average	37.5	132	612	0.74
(nmol/L)	CV	4.6	3.0	2.6	
XA	Average	17.2	76.7	378	0.55
(nmol/L)	CV	6.1	3.1	3.5	
QUIN	Average	342	1492	7779	2.5
(nmol/L)	CV	3.8	4.0	2.8	
PA	Average	29.2	157	718	1.9
(nmol/L)	CV	5.6	3.6	3.2	
NA	Average	11.8	59.3	365	1.7
(nmol/L)	CV	14.3	6.7	4.9	
ANA	Average	51.7	253	1565	5.5
(nmol/L)	CV	13.6	6.3	3.6	

Abbreviations: QC = quality control; LOQ = limit of quantification, 3-HK = 3-hydroxykynurenine, KYN = kynurenine, TRP = tryptophan, KYNA = kynurenic acid, XA = xanthurenic acid, QUIN = quinolinic acid, PA = picolinic acid, NA = nicotinic acid, ANA = anthranilic acid.
